# Supplementary material for: Effects of DPP4 Inhibitor in Platelet Reactivity and Other Cardiac Risk Markers in Patients with Type 2 Diabetes and Acute Myocardial Infarction
Source: J Clin Med. 2022 Sep 29;11(19):5776. doi: 10.3390/jcm11195776 (PMC9571017; doi:10.3390/jcm11195776)
Supplement: Supplementary file 1 [file jcm-11-05776-s001.zip › jcm-1892929-Supplementary.pdf]

Effects of DPP4 inhibitor in platelet reactivity and other cardiac risk markers in patients with type 2 diabetes and acute myocardial infarction

Supplementary material

Inclusion criteria

1) Men and women aged ≥ 18 years (women should be postmenopausal or surgically sterilized); 2) acute myocardial infarction, with or without ST segment elevation (STEMI/NSTEMI) according to the 3rd Universal Definition of Acute Myocardial Infarction,[14], with up to 72 hours of evolution from the onset of symptoms and in use of dual antiplatelet therapy (DAPT) with acetylsalicylic acid (aspirin™) plus a P_2Y_{12} receptor inhibitor; 3) previous diagnosis of type 2 DM according to criteria of the American Diabetes Association, in chronic use of antihyperglycemic medication; and/or patients without previous diagnosis of DM, but with glycated hemoglobin ≥ 6.5% in current hospitalization.[15]

Exclusion criteria

1) Estimated glomerular filtration rate (e-GFR) < 30 ml/min/1.73 m^2, calculated by the Modification of Diet in Renal Disease (MDRD) formula, or prior renal transplant; 2) Body mass index (BMI) ≥ 50 Kg/m^2; 3) patients in current use of corticosteroid therapy and/or within 14 days prior to hospitalization; 4) patients with clinical classification of Killip>2 at randomization; 5) patients in septic shock or severe glycemic decompensation requiring the use of insulin infusion at randomization; 6) any of the cardiovascular conditions below within 12 weeks before the current hospitalization: AMI, HF functional class NYHA > 2, percutaneous coronary intervention (PCI), surgery for myocardial revascularization or stroke; 7) patients using enteral or parenteral nutrition; 8) patients using any GLP-1 receptor agonists or DPP4 inhibitors in the last 6 months; 9) patients using CYP P450 3A4 and 3A5 inhibitors; 10) patients with prior diagnosis of pancreatitis of any etiology and at any time; 11) patients undergoing bariatric surgery in the last 2 years and/or other gastrointestinal surgeries that induce chronic malabsorption; 12) blood dyscrasias or any disorder that causes hemolysis, previously known; 13) known thrombophilia or thrombocytosis; 14) hematocrit > 50% or < 30%; 15) any medical condition which investigator's opinion presents significant risk to the patient or interferes with the interpretation of safety and efficacy data; 16) patients who are participating in another clinical study.

Procedures for platelet reactivity analysis

To assess platelet reactivity by VerifyNow Aspirin™ blood sample were collected in 2.0 ml tubes containing 3.2% citrate (Vacutainer™) and transferred to containers with fibrinogen and arachidonic acid (AA), where it was made analysis of the response to aspirin.™ As aggregation occurs, the system converts the transmitted luminosity into Aspirin Reaction Units (ARU). The higher reactivity, means higher result in ARU. In the Multiplate Aspirin™ and ADP™ (Roche Diagnostics, Rotkreuz, Switzerland) test, blood sample is previously collecteded in 3.0 ml tubes containing hirudin. Evaluation of reactivity was made by electrical impedance between two electrodes immersed in total blood 6 minutes after the addition of the agonist. The increase in impedance correlates with the amount of platelet aggregates that are deposited in the electrodes after the addition of the agonist. ADP agonists and arachidonic acid were used to evaluate the effect respectively of P2Y12 receptor inhibitor and aspirin™. As the reaction occurs, a graph of electrical impedance is drawn according to the reaction time, so that reactivity is evaluated by the area under the curve (AUC), where the higher the AUC, the greater the reactivity for the reagent used. To ensure the quality control all steps were carried out with the following procedure: blood samples were collected from the antecubital vein in supine position

using vaccum tubes, with a large bore needle (19G) with maximal care to avoid local trauma or stasis during venopuncture; the initial blood drawn after venopuncture was discarded (few mililiters), and immediately the sample was mixed five times with the anticoagulant before performing the analysis. In both tests, blood samples were analyzed between 30 and 240 minutes after collection, according to the manufacturer's recommendations. Platelet function tests were performed between 2 to 24 hours after last aspirin intake (just before the next drug administration).

Procedures for Continuous glucose monitoring (CGM) analysis

This system consists of a device recorder (IPro-2™) connected to a sensor (Enlite™) implanted in subcutaneous fat tissue in the abdominal region, where interstitial glucose is measured by an electrochemical assay and calibrated with point-of-care capillary blood glucose. Every ten seconds a glucose measurement is taken, and every 5 minutes, an average value is recorded in the device's memory (288 measurements per day). Glucose metrics were obtained off-line at hospital discharge, when the device was removed and data transferred to a software for pre-specified analisys (mean, standard deviation, MAGE, and hypoglycemic events)

Results

Figure S1: Effects of DPP-4i on platelet reactivity (Mean ± SD) by Multiplate Aspirin.

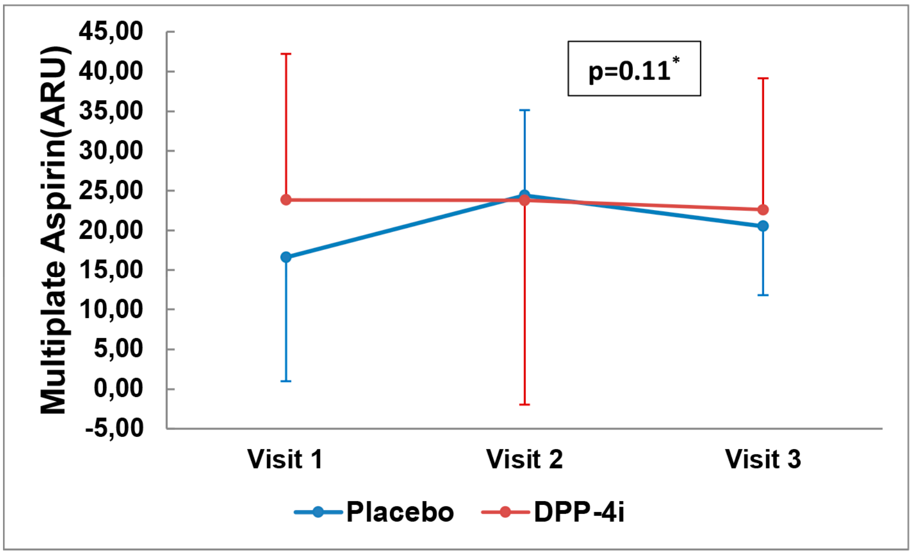

Visit 1: baseline   Visit 2: 4 ± 2 days   Visit 3: 30 ± 5 days

Visit 1: Placebo (16.62 ± 15.63); DPP-4i (23.84 ±18.39)

Visit 2: Placebo (24.42 ± 10.73); DPP-4i (23.80 ± 25.77)

Visit 3: Placebo (20.54 ± 8.72); DPP-4i (22.60 ± 16.55)

* ANOVA test for repeated measures   values expressed as AUC: area under curve

Figure S2: Effects of DPP-4i on platelet reactivity (Mean ± SD) by Multiplate ADP.

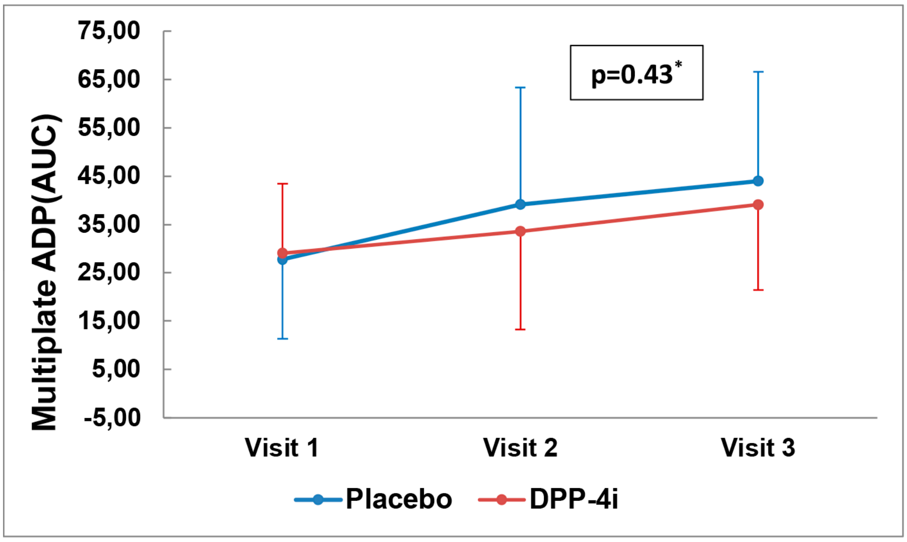

Visit 1: baseline   Visit 2: 4 ± 2 days   Visit 3: 30 ± 5 days

Visit 1: Placebo (27.81 ± 16.51); DPP-4i (29.12 ±14.35)

Visit 2: Placebo (39.19 ± 24.21); DPP-4i (33.60 ± 20.39)

Visit 3: Placebo (44.00 ± 22.61); DPP-4i (39.12 ± 17.68)

* ANOVA test for repeated measures   values expressed as AUC: area under curve

Figure S3: Non-inferiority analysis for BNP between groups.

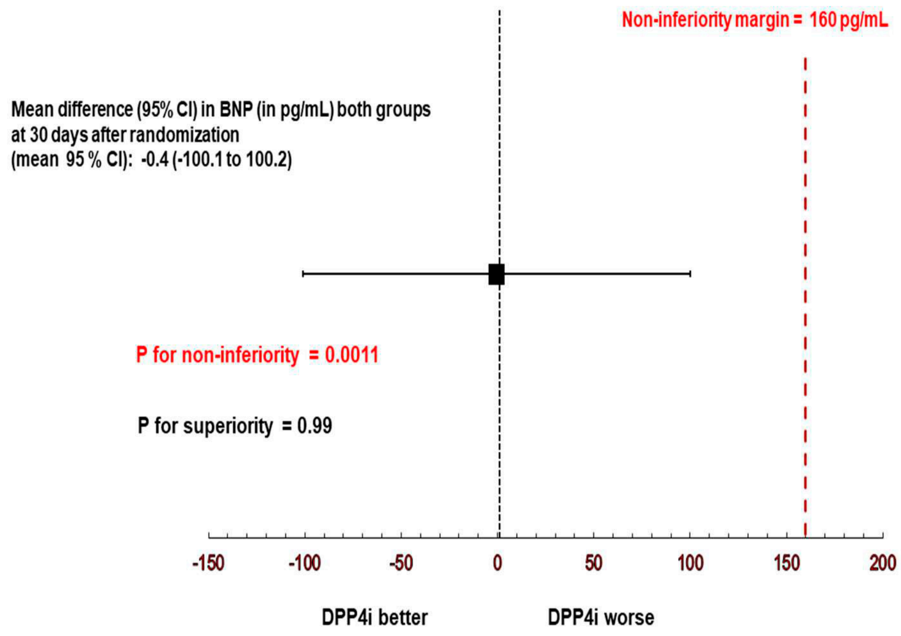

Figure S4(a): Changes in hs-CRP (Δ).

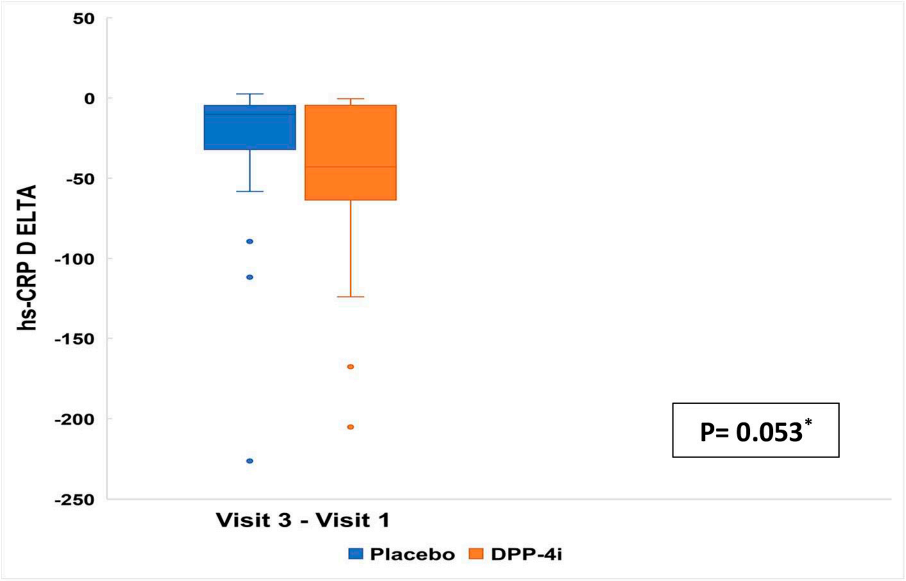

Visit 1: baseline Visit 3: 30 ± 5 days

Placebo: - 10.26 (-26.99; - 4.70); DPP-4i: - 42.88 (- 63.60; -4.56)

hs-CRP=high sensitive C-reactive protein *Mann-Whitney test

Figure S4(b): Changes in hs-CRP (Δ) without outliers.

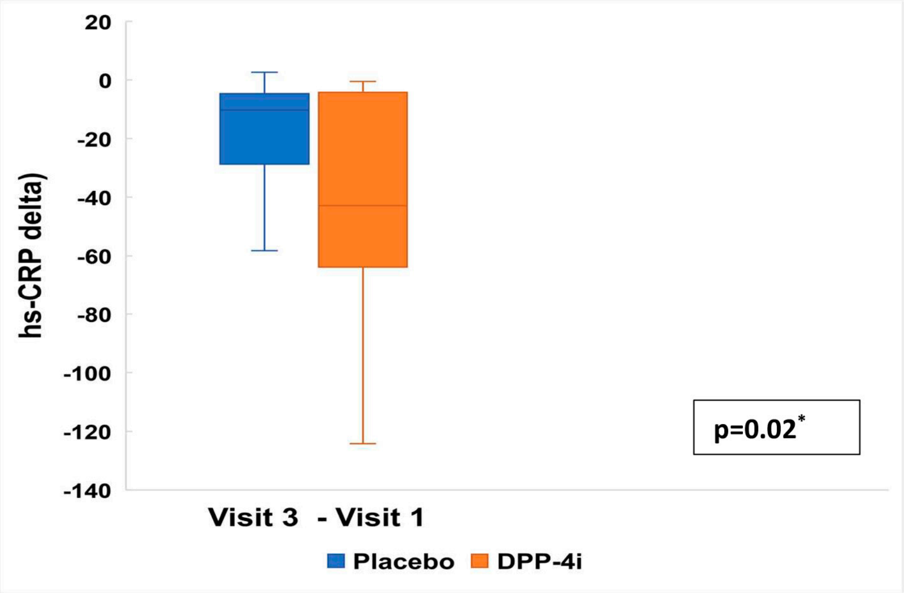

Visit 1: baseline   Visit 3: 30 ± 5 days

Placebo: - 8.62 (-16.69; - 4.64); DPP-4i: - 25.96 (- 50.65; -3.94)

hs-CRP=high sensitive C-reactive protein   *Mann-Whitney test

Table S1. Medications before hospital admission.

	Overall (n=70)	Placebo (n=35)	DPP-4i (n=35)
ASA, n(%)	24 (34.8%)	12 (34.3%)	12 (34.3%)
Clopidogrel, n(%)	6 (8.6%)	4 (11.4%)	2 (5.7%)
Statin, n(%)	31 (44.9%)	17 (48.6%)	14 (41.2%)
Fibrates	1(1.4%)	0 (0.0%)	1 (2.9%)
Betablockers, n(%)	24 (34.8%)	15 (42.9%)	9 (26.5%)
Diuretics, n(%)	14 (20.0%)	11 (31.4%)	3 (8.6%)
ACEi/ARB, n(%)	44 (62.9%)	23 (65.7%)	21 (60.0%)
MRA, n(%)	1 (1.4%)	1 (2.9%)	0 (0.0%)
CCB, n(%)	14 (20.0%)	8 (22.9%)	6 (17.1%)
Sulfonylurea, n(%)	27 (38.6%)	17 (48.6%)	10 (28.6%)
Metformin, n(%)	57 (81.4%)	28 (80.0%)	29 (82.9%)
Basal Insulin, n(%)	15 (21.4%)	6 (17.1%)	9 (25.7%)
SGLT2-i, n(%)	4 (5.7%)	3 (8.6%)	1 (2.9%)
TZD, n(%)	1 (1.4%)	0 (0.0%)	1 (2.9%)

ASA= acetylsalicylic acid; ACEi/ARB= angiotensin converting enzyme inhibitors/angiotensin II receptor blockers; MRA= mineralocorticoid receptor antagonist; CCB= calcium channel blockers; SGLT2-i= sodium glucose co-transporter type 2 inhibitors; TZD= glitazones. All p-values for comparisson between DPP-4i and placebo were non significant.

Table S2. Cardiovascular medications for AMI.

	Overall (n=70)	Placebo (n=35)	DPP-4i (n=35)
Statin, n(%)	70 (100.0%)	35 (100.0%)	35 (100.0%)
Betablockers, n(%)	56 (80.0%)	28 (80.0%)	28 (80.0%)
ACEi , n(%)	55 (78.6%)	29 (82.9%)	26 (74.3%)
ARB, n(%)	11 (15.7%)	5 (14.3%)	6 (17.1%)
MRA, n(%)	14 (20.0%)	7 (20.0%)	7 (20.0%)
CCB, n(%)	5 (7.1%)	3 (8.6%)	2 (5.7%)
GPIIb/IIIa inhibitor,n(%)	6 (8.6%)	5 (14.3%)	1 (2.9%)
Nitrates, n(%)	8 (11.4%)	4 (11.4%)	4 (11.4%)
Diuretics, n(%)	6 (8.5%)	3 (8.5%)	3 (8.5%)
LMWH, n(%)	69 (98.6%)	34 (97.1%)	35 (100.0%)
PPI, n(%)	18 (25.7%)	6 (17.1%)	12 (34.3%)
H_2 inhibitor, n(%)	50 (71.4%)	29 (82.9%)	21 (60.0%)

ACEi= angiotensin converting enzyme inhibitors; ARB= angiotensin II receptor blockers; MRA= mineralocorticoid receptor antagonist; CCB= calcium channel blockers; LMWHS= low-molecular-weight heparins; PPI= proton pump inhibitor. All p-values for comparison between DPP4i and placebo non-significant, except use of H2 inhibitor(p=0.03).

Table S3. Subgroup analysis: Mean (±SD) of PR by VerifyNow Aspirin between control and intervention groups in each subgroup for primary objective.

Subgroup		Visit 1	Visit 2	p *
BMI < 30 kg/m²	Placebo (n=21)	493.05 (63.62)	464.57 (61.11)	0.58
	DPP-4i (n=29)	472.14 (72.49)	457.10 (54.90)	
BMI ≥ 30 kg/m²	Placebo (n=14)	475.07 (52.34)	476.93 (69.80)	0.09
	DPP-4i (n=6)	435.50 (34.70)	501.50 (38.57)	
Female sex	Placebo (n=14)	482.14 (66.18)	467.29 (64.42)	0.79
	DPP-4i (n=11)	469.18 (68.24)	464.00 (52.52)	
Male sex	Placebo (n=21)	488.33 (55.68)	471.00 (65.28)	0.47
	DPP-4i (n=24)	464.33 (70.12)	465.04 (56.74)	
Age < 65 ys-old	Placebo (n=22)	489.82 (59.40)	480.23 (69.18)	0.64
	DPP-4i (n=22)	454.77 (61.87)	457.59 (59.13)	
Idade ≥ 65 ys-old	Placebo (n=13)	479.15 (60.72)	451.38 (51.62)	0.52
	DPP-4i (n=13)	484.62 (77.54)	476.77 (45.83)	
Non-smoking	Placebo (n=25)	479.36 (58.70)	476.08 (64.39)	0.96
	DPP-4i (n=23)	461.96 (60.10)	459.70 (61.84)	
Smoking	Placebo (n=10)	502.10 (60.43)	453.10 (63.25)	0.16
	DPP-4i (n=12)	473.33 (84.92)	474.33 (37.94)	
Time of diagnosis of T2DM < 9 ys	Placebo (n=18)	492.50 (55.76)	467.44 (72.36)	0.15
	DPP-4i (n=16)	476.19 (75.31)	496.13 (43.88)	
Time of diagnosis of T2DM ≥ 9 ys	Placebo (n=17)	478.82 (63.62)	471.71 (55.97)	0.65
	DPP-4i (n=19)	457.16 (63.07)	438.26 (49.26)	
Baseline glucose < 125 mg/dL	Placebo (n=8)	478.25 (56.13)	523.88 (56.08)	-
	DPP4-i (n=1)	-	-	
Baseline glucose ≥ 125 mg/dL	Placebo (n=27)	488.11 (60.96)	453.41 (57.73)	0.10
	DPP-4i (n=34)	465.06 (69.42)	464.91 (55.49)	
HbA1c < 9%	Placebo (n=9)	487.00 (67.60)	476.33 (71.79)	0.79
	DPP-4i (n=10)	458.00 (62.45)	458.90 (71.25)	
HbA1c ≥ 9%	Placebo (n=26)	85.46 (57.51)	467.15 (62.45)	0.48
	DPP-4i (n=25)	469.00 (71.83)	467.04 (48.08)	
AMI treatment PCI and/or pharmac.trombolysis	Placebo (n=34)	487.88 (58.87)	471.65 (63.69)	0.30
	DPP4 (n=33)	457.61 (61.36)	462.35 (55.31)	
Medical only	Placebo (n=1)	-	-	-
	DPP4 (n=2)	-	-	

* ANOVA test for repeated measures BMI= body mass index; AMI= acute myocardial infarction; T2DM= type 2 diabetes mellitus; HbA1c= glycated hemoglobin; PCI= percutaneous coronary intervention; PR= platelet reactivity.

Table S4. Differences in PR (mean ±SD) expressed by ARU by VerifyNow Aspirin between placebo and each DPP-4i subgroup.

	Placebo (n=34)	Sitagliptin (n=19)	Saxagliptin (n=14)
Visit 1	486.45 ± 61.00	463.79 ± 65.59	454.77 ± 69.45
Visit 2	469.94 ± 65.77	455.68 ± 49.36	474.38 ± 66.54
Visit 3	459.27 ± 54.50	440.89 ± 46.04	440.54 ± 70.57

* ANOVA test for repeated measures PR= platelet reactivity ARU= aspirin reaction units p=0.80 (ANOVA test)

Table S5. Glycemic control and insulin therapy.

	Overall (n=70)	Placebo (n=35)	DPP-4i (n=35)	p-value
Mean(±SD) BG	181.81± 6.35	179.03 ± 4.75	184.59 ± 48.39	0.61 [a]
Number of any BG reading < 70mg/d, n(%)	4 (5.7%)	1 (2.9%)	3 (8.6%)	0.61 [c]
Number of pats with supplemental (rapid-action) insulin, n(%)	58 (82.9%)	27 (77.1%)	31 (88.6%)	0.20 [b]
Total supplemental (rapid-action) insulin dose, Units/day(median-IIQ)	16.00 (4.00; 26.00)	16.00 (0.00; 26.00)	18.00 (4.00; 34.00)	0.41 [d]
Number of pats with basal (long-action) insulin, n(%)	17 (24.3%)	9 (25.7%)	8 (22.9%)	0.78 [b]

BG= capillary blood glucose concentration (mg/dL; pats= patients; [a] Student t test; [b]Chi square; [c]Fisher exact test; [d] Mann-Whitney test.

Table S6. Glycemic control evaluated by CGM.

	Overall (n=27)	Placebo (n=15)	DPP-4i (n=12)	p
Mean(±SD)*	162.74 ± 5.53	167.90 ±45.00	156.30 ± 47.34	0.52 [a]
Coefficient of variation (%) mean(±SD)	21.94 ± 7.79	20.65 ± 7.44	23.70 ± 8.25	0.33 [a]
% TIR> 180 mg/dL median(IIQ)	16.83 (2.63; 61.21)	34.89 (1.04; 62.41)	15.92 (4.72; 36.80)	0.85 [b]
% TIR< 70 mg/dL median(IIQ)	0.00 (0.00; 0.77)	0.00 (0.00; 0.77)	0.00 (0.00; 1.36)	0.95 [b]
MAGE * median(IIQ)	110.53 (64.38; 140.89)	101.57 (63.22; 33.63)	120.68 (84.89; 175.87)	0.42 [b]

* mg/dL; CGM= continuous glucose monitoring; TIR= time in range; MAGE= mean amplitude of glycemic excursions [a] Student t test; [b] Mann-Whitney test.

Table S7. Differences in BNP* (mean ± SD) between placebo and each DPP-4i subgroup.

	Placebo (n=34)	Sitagliptin (n=19)	Saxagliptin (n=14)
Visit 1	194.12 ± 197.81	156.00 ± 144.33	279.07 ± 186.53
Visit 3	180.03 ±161.11	124.74 ± 8.,66	254.00 ± 349.07

*pg/mL p=0.54 (ANOVA test for repeated measures)

Table S8. Differences in ALT (mean ± SD) between placebo and DPP-4i.

	Group	
ALT*	Placebo (n=33)	DPP-4i (n=33)
Visit 1	48,91 ±20,27	52,76 ± 39,56
Visit 3	35,64 ± 26,53	34,94 ± 20,18

p=0,82 (ANOVA test for repeated measures)

(*) ALT: Alanine aminotransferase expressed as U/L

Table S9. Differences in AST (mean ± SD) between placebo and DPP-4i.

	Group	
AST*	Placebo (n=33)	DPP-4i (n=33)
Visit 1	113,35 ± 150,07	149,09 ± 170,62
Visit 3	24,06 ± 11,68	22,03 ± 8,56

p=0,15 (ANOVA test for repeated measures)

(*) AST: Aspartate aminotransferase expressed as U/L

Table S10. Differences in Amylase (mean ± SD) between placebo and DPP-4i.

	Group	
Amylase	Placebo (n=34)	DPP-4i (n=33)
Visit 1	56,35 ± 24,14	50,64 ± 19,53
Visit 3	68,35 ± 23,10	70,30 ± 25,60

p=0,11 (ANOVA test for repeated measures)

(*) Amylase expressed as U/L

Table S11. Differences in Lipase (mean ± SD) between placebo and DPP-4i.

	Group	
Lipase*	Placebo (n=34)	DPP-4i (n=33)
Visit 1	142,47 ± 64,90	155,18 ± 111,20
Visit 3	209,85 ± 106,57	211,05 ± 139,69

p=0,56(ANOVA test for repeated measures)

(*) Lipase expressed as U/L

Table S12. Differences in CK-Mb peak between placebo and DPP-4i.

		Group		
	Overall (n=70)	Placebo (n=35)	DPP-4i (n=35)	p
CK-MB ng/mL(*)	104,92 ± 112,01	81,51 ± 4,15	128,32 ± 124,38	0,11[(1)]

(1) Student t-test

Table S13. Incidence of adverse events (30-days follow-up).

	Overall (n=70)	Placebo (n=35)	DPP-4i (n=35)	p-value
AE(global), n(%)	34 (48.6%)	19 (54.3%)	15 (42.9%)	0.33 [a]
SAE, n(%)	5 (7.0%)	2 (5.7%)	3 (8.6%)	1.00 [b]
AMI	1	0	1	
HFH	1	0	1	
UA	2	2	0	
Dead	1	0	1	

AE= adverse event; SAE= serious adverse event; AMI= acute myocardial infarction; HFH= heart failure hospitalization; UA= unstable angina. [a] Chi square test; [b] Fisher exact test.

Table S14. Incidence of adverse events (30-days follow-up) between placebo and each DPP-4i subgroup.

	Overall (n=70)	Placebo (n=35)	Saxagliptin (n=15)	Sitagliptin (n=20)
AE(global), n(%)	34 (48.6%)	19 (54.3%)	10 (66.7%)	5(33.3%)
SAE, n(%)	5 (7.0%)	2 (5.7%)	3 (20.0%)	0
AMI	1	0	1	0
HFH	1	0	1	0
UA	2	2	0	0
Dead	1	0	1	0

AE= adverse event; SAE= serious adverse event; AMI= acute myocardial infarction; HFH= heart failure hospitalization; UA= unstable angina
